# ID 333 - Mapeamento da Periodicidade da Atualização das Diretrizes Clínico-Assistenciais do SUS

**DOI:** 10.5327/2237-9622.2025.v34s1.333

**Published:** 2025-11-25

**Authors:** Maíra Barroso Pereira, Adriane Lopes Medeiros Simone, Rafaela Alves Padua, Guilherme de Almeida Moraes, Edwirgens Mariana Albuquerque, Tamiê de Camargo Martins, Tainá Freitas Saldanha, Andreia Ramos Lira, Andréa da Silva Dourado, Stéfani Sousa Borges, Daniela Oliveira de Melo

**Keywords:** diretrizes clínico-assistenciais, guia de prática clínica, protocolos clínicos, priorização, Sistema Único de Saúde

## Abstract

**Introdução:**

As diretrizes clínico-assistenciais (DCA) apresentam recomendações para o cuidado em saúde, desempenhando também um papel importante na gestão e na regulação dos sistemas de saúde. Endossadas pela Comissão Nacional de Incorporação de Tecnologias no Sistema Único de Saúde (Conitec), as DCA do Ministério da Saúde (MS) são publicadas como: Protocolos Clínicos e Diretrizes Terapêuticas (PCDT), Diretrizes Diagnósticas e Terapêuticas (DDT), Protocolos de Uso (PU) ou Diretrizes Nacionais (DN), devendo ser atualizadas a cada dois anos (antes de dezembro de 2022) ou quando da incorporação, alteração ou exclusão de tecnologias no SUS e da existência de novas evidências científicas (a partir da publicação do Decreto n.º 11.161, de 4 de agosto de 2022). Este estudo teve como objetivo mapear a periodicidade de atualização e compreender elementos sobre o processo de priorização de temas para elaboração e atualização das DCA pelo MS.

**Método:**

Trata-se de um estudo descritivo, baseado nas DCA vigentes disponíveis no portal da Conitec em 30 de agosto de 2024. Os dados foram coletados utilizando formulário específico (número da portaria vigente e a data de publicação, como também das portarias revogadas, quando pertinente) e analisados por meio de estatística descritiva. Os documentos foram classificados em primeira publicação ou atualização, por tipo de DCA do MS e área de especialidade (doenças raras, oncologia e outras). Foram analisados os intervalos, em anos, da primeira publicação e as respectivas atualizações.

**Resultados:**

Foram analisadas 174 DCA, que datavam entre 2007 e 2024, sendo mais frequentes as publicações em 2022 (14,9%), 2018 (13,8%) e 2021 (13,8%). Do total, 71 (40,8%) eram a primeira publicação e 103 (59,2%) eram atualizações, sendo os PCDT o tipo de DCA com maior percentual (74,1%) de atualizações no período. Entre as atualizações, 31,1% eram para doenças raras, 16,5% para oncologia e 52,4% para outras condições. O tempo mediano entre a primeira publicação e a primeira atualização foi de seis anos, com intervalo interquartil de 2,9 a 7,9 anos, em que 25% das DCA foram atualizadas em até 2,9 anos, 50% entre 2,9 e 7,9 anos, e 25% após 7,9 anos. O tempo mediano das DCA para doenças raras (n=32) foi de 6,7 anos, e para oncologia (n=17) de 7,3 anos, com intervalo interquartil de 5,3 a 8,1 anos e de 4,7 a 9 anos, respectivamente.

**Conclusão:**

A mudança na legislação, a partir de dezembro de 2022, foi oportuna, adequando o processo de atualização das DCA à realidade observada, visto que a maioria dos documentos não eram atualizados a cada dois anos. Entre as razões, a ausência de necessidade (sem incorporação de um novo tratamento ou novas evidências sobre a condição de saúde) e fragilidades ou barreiras na gestão do processo de atualização, como a escassez de Núcleos de Avaliação de Tecnologias em Saúde (Nats) qualificados para construção de DCA. Atualmente, o MS utiliza parâmetros além do tempo de publicação da DCA para priorização da atualização, como o Monitoramento do Horizonte Tecnológico, já adotado por outros países, como o Reino Unido, o Canadá e a Austrália. O desenvolvimento de uma DCA é um processo rigoroso e complexo, que demanda esforços e recursos consideráveis. Os resultados apontam para a necessidade de um planejamento baseado em critérios de priorização, para que o processo ocorra de forma transparente, eficiente, equânime e sustentável.

